# Areca nut extracts mobilize calcium and release pro-inflammatory cytokines from various immune cells

**DOI:** 10.1038/s41598-017-18996-2

**Published:** 2018-01-18

**Authors:** Malika Faouzi, Ram P. Neupane, Jian Yang, Philip Williams, Reinhold Penner

**Affiliations:** 1 Center for Biomedical Research, The Queen’s Medical Center, Honolulu, HI 96813 USA; 2 Department of Chemistry, University of Hawaii, Honolulu, HI 96822 USA; 3 College of Natural and Applied Sciences, University of Guam, Mangilao, 96923 Guam USA; 4 Cancer Biology Program, University of Hawaii Cancer Center, Honolulu, HI 96813 USA

## Abstract

Betel nut consumption has significant implications for the public health globally, as the wide-spread habit of Areca chewing throughout Asia and the Pacific is associated with a high prevalence of oral carcinoma and other diseases. Despite a clear causal association of betel nut chewing and oral mucosal diseases, the biological mechanisms that link Areca nut-contained molecules, inflammation and cancer remain underexplored. In this study we show that the whole Areca nut extract (ANE) is capable of mobilizing Ca^2+^ in various immune cell lines. Interestingly, none of the four major alkaloids or a range of other known constituents of Areca nut were able to induce such Ca^2+^ signals, suggesting that the active components might represent novel or so far unappreciated chemical structures. The separation of ANE into aqueous and organic fractions has further revealed that the calcium-mobilizing molecules are exclusively present in the aqueous extract. In addition, we found that these calcium signals are associated with the activation of several immune cell lines as shown by the release of pro-inflammatory cytokines and increased cell proliferation. These results indicate that calcium-mobilizing molecules present in the aqueous fraction of the Areca nut may critically contribute to the inflammatory disorders affecting betel nut chewers.

## Introduction

Betel or Areca nuts are used around the world by approximately 600 million people and are ranked fourth in worldwide use among psychoactive substances. It has been clearly established that the chewing of betel quid causes oral lesions and pathological epidermal changes within the mouth that potentiate malignant transformations and can lead to the development of esophageal and oral cancers^1,2^. The habit is also implicated in other diseases, including liver cirrhosis, hepatocellular carcinoma, obesity, hypertension, type 2 diabetes, chronic kidney disease, hyperlipidemia, and metabolic syndrome^3–5^. Additional studies have shown betel nut use also causes cardiovascular disease^6^, aggravates asthma^7^, and can affect reproductive health^8,9^. In addition to it being a global health issue across Asia and the pacific islands, betel nut consumption must also be considered in the context of health disparities, both in Guam and Hawai’i, as the habit is most prevalent in American minority populations such as Chamorros and Micronesian people in Guam and Hawai’i^10–12^.

Despite a clear causal association of betel nut chewing and oral mucosal diseases such as leukoplakia, oral submucous fibrosis and oral cancer^4,13,14^, there remain significant knowledge gaps in the understanding of the underlying mechanisms. Various compounds have been isolated and identified from *Areca catechu*, including alkaloids, tannins, flavones, triterpenes, steroids, and fatty acids. Extracts or compounds isolated from Areca nuts have many pharmacological activities that range from beneficial medicinal purposes to serious health risks^15^. The carcinogenicity of various Areca nut components has been the subject of numerous studies using both *in vitro* and *in vivo* assays^4,15,16^. Polyphenols and tannins contained in Areca nuts have been reported to exert both carcinogenic and anti-carcinogenic effects^14,16,17^. Similarly, Areca alkaloids have been demonstrated to have mutagenic and genotoxic effects in many short-term assays^14,15,18^, but their genotoxicity to oral fibroblasts and keratinocytes, the target cells of betel nuts, has not been identified. It would thus appear that Areca nut toxicity is not completely due to its polyphenol, tannin or alkaloid content and further factors may be contributing.

Reactive oxygen species produced during auto-oxidation of polyphenols in the betel nut chewer’s saliva as well as the nitrosation of alkaloids, favored by the presence of slaked lime (commonly added to betel nut preparations), appear to be important in the initiation and promotion of oral cancer^14–16^. Finally, Areca nut chewing also promotes the release of various mediators from host cells that contribute to a chronic inflammatory microenvironment in the oral cavity and further supports the development of oral lesions and tissue damage. It is now widely acknowledged and appreciated that chronic inflammation plays an important role in carcinogenesis, but the biological mechanisms that link Areca nut-contained molecules, immune cell activation, cytokine production, inflammation, and cancer remain underexplored and are the focus of the present study.

Inflammation is a complex immunological response in tissues experiencing harmful stimuli^19–23^. It involves the migration of several immune cells from the vasculature into damaged tissues to remove the agents that cause tissue injury and help remodel the area. The process of acute inflammation is a limited response that is commonly initiated by resident mast cells, dendritic cells, and monocytes/macrophages, and followed by the infiltration of mostly polymorphonuclear leukocytes (PMN). After 24 to 48 hours, monocytes predominate and begin to differentiate into macrophages, which then attract lymphocytes. If this process remains unresolved, it can lead to chronic inflammation and severe tissue damage. Chronic inflammation is characterized by the abundance of monocytes, macrophages, and lymphocytes, creating an environment that favors the production of pro-inflammatory cytokines and reactive oxygen species, which in turn establishes favorable conditions for transformation and growth of cancer cells.

Exposure to betel nut components that promote immune cell activation has the potential to establish a pro-inflammatory environment that could initiate cancer and/or exacerbate the inflammation in support of existing neoplasms. Although anecdotal anti-inflammatory effects of Areca nut extracts have been reported^24,25^, the preponderance of available data has shaped the general consensus that pro-inflammatory mechanisms are major factors contributing to the increased risk of periodontal disease, oral submucous fibrosis, and oral squamous cell carcinoma associated with Areca chewing^4,5,26–28^. The host immune responses triggered by Areca nuts may establish an inflammatory environment in the oral cavity that can initiate and/or contribute to the above diseases. In addition to ROS, the tissue destruction in periodontal disease mainly results from various pro-inflammatory mediators^27,29,30^. A previous study has found that incubation of polymorphonuclear leukocytes with Areca nut extracts resulted in the phosphorylation of p38 MAPK and Ca^2+^ mobilization, resulting in the release of pro-inflammatory lipid mediators such as leukotriene B4^30^. However, it remains unclear what the source of calcium is, which leukocytes (neutrophils, basophils, eosinophils, mast cells) are responsive and whether other immune cells, including those of the adaptive immune system, are activated by Areca nut extracts. In this study, we demonstrate that Areca extracts are capable of inducing cytokine production by mobilizing Ca^2+^ in various cells of the innate and adaptive immune system (T lymphocytes, mast cells, and monocytes), which can contribute to chronic inflammation and potentially play an important role in oral diseases of betel nut chewers.

## Materials and Methods

### Biological Material and Chemicals

Young *Areca catechu* nuts and *Piper betle* leaves were obtained from a local convenience store in Honolulu. The Areca nuts were cut into halves, scooped out of the husks, and freeze-dried for storage. All the chemicals tested in calcium bio-assays were purchased from Sigma-Aldrich (St. Louis, Missouri). The immune cell lines were obtained from ATCC (Manassas, Virginia). RBL-2H3 cells expressing the Muscarinic Acetylcholine receptor M1 were a kind gift from Dr. Michael Beaven^31^. Primary human Peripheral Blood Mononuclear Cells (PBMCs) were purchased from Lonza (Walkersville, Maryland) and Stemcell Technologies (Tukwila, Washington).

### Cell culture

Jurkat, U937, HL-60 cell lines and PBMCs were maintained in RPMI 1640 medium (Clontech) supplemented with 10% fetal bovine serum (FBS). RBL-2H3 cells (WT and M1) were cultured in DMEM medium (Clontech) with 10% fetal bovine serum (FBS). All cells were cultured at 37 °C, 5% CO_2_, and 95% humidity.

### Extraction Procedure

Chopped young, wet *Areca catechu* nuts (~10 g each) were extracted three times (25 ml each) in acetone, dichloromethane, methanol, ethanol or a mixture of chloroform, methanol and water (12:5:3). Solvent was removed *in vacuo*, affording 1.623 g, 0.132 g, 1.560 g, 1.719 g and 1.656 g of crude extract, respectively. The crude extracts were dissolved in 6 ml of aqueous methanol (1:1) and partitioned three times (6 ml each) with chloroform. Upon removal of solvent, aqueous and chloroform fractions of each crude extract were obtained. Based on Ca^2+^-mobilizing activity data of crude extracts and their fractions in three different cell lines (Jurkat, U937 and RBL-2H3), acetone was chosen as the optimal solvent for further extractions.

Areca nut husks (~20 g) and *Piper betle* leaves (~4 g) were also extracted three times (25 ml each) in a mixture of chloroform, methanol and water (12:5:3). Solvent was removed from the combined extracts to afford 0.276 g and 0.352 g of crude husk extract and crude leaf extract, respectively.

For the separation of aqueous and chloroform fractions, 3.051 g of the lyophilized Areca nut extract was dissolved in water (50 ml), partitioned three times (50 ml each) with chloroform, and the solvent evaporated, affording 2.445 g of aqueous extract and 0.459 g of chloroform extract.

### Fura-2AM based Calcium Bioassay

The effect of various extracts and chemicals was assessed using a high-throughput kinetic plate reader instrument (Hamamatsu FDSS-7000EX). Cells that grow in suspension (Jurkat, U937, HL-60 and PBMCs) were incubated with the loading medium for one hour, washed with bath solution and plated in 96-well plates at a density of 100,000 cells/well before proceeding with Ca^2+^ measurement. Adherent cells RBL-2H3 (WT and M1) were pre-plated overnight at a density of 60,000 cells/well to let the cells adhere before fura-2 loading and Ca^2+^ measurement on the following day. The loading medium was composed of the culture medium supplemented with 2 mM Probenecid, 0.1% Pluronic and 2 µM Fura-2-AM. Bath solution contained in mM: 140 NaCl, 0 or 2 CaCl_2_, 2 MgCl_2_, 10 glucose, and 10 Hepes (pH 7.2 with NaOH). Stock solutions of extracts and all the tested chemicals were prepared in DMSO. DMSO control was then included in all experiments to monitor the solvent effect.

### Cytokine release Assay

Jurkat, U937 and HL-60 cells were plated in 24-well plates at a density of 10^6^ cells/well and induced for 24 h. Cells were then pelleted and the supernatants were transferred into Eppendorf tubes and assayed for cytokine release, using the Cytokine Human 10-Plex Kit (Invitrogen). This kit is designed for the Luminex™ platform and allows quantifying the amounts of human GM-CSF, IFN-γ, IL-1β, IL-2, IL-4, IL-5, IL-6, IL-8, IL-10 and TNF-α in the cell culture supernatants. Cytokine release following the exposure to Areca nut extracts (50 µg/ml) was compared to the untreated cells and cells treated with the vehicle (DMSO) only. The positive controls for calcium-induced cytokine release are as follows: Jurkat cells were stimulated using 50 ng/ml *phorbol* 12-myristate 13-acetate (PMA) and 1 µM ionomycin, U937 cells were stimulated with 10 ng/ml PMA and 20 µg/ml Lipopolysaccharides (LPS), and HL-60 cells were stimulated with 10 ng/ml PMA.

### Cell growth Assay

In order to generate the conditioned medium, we plated Jurkat cells in 24-well plates at a density of 2 × 10^6^ cells/ml/well and incubated them overnight in the culture medium alone or supplemented with 0.2% DMSO (vehicle) or different AN extracts (at 50 µg/ml final concentration). On the following day, cells were pelleted to remove the supernatants containing the treatments, re-suspended in fresh culture medium then incubated for 24 h to let the cytokine release occur. After the 24 hours incubation, cells were pelleted and the supernatants were collected in sterile tubes. For the growth assay, Jurkat, U937 and HL-60 cell lines were suspended in different conditioned mediums and plated in 96-well plates at a density of 2 × 10^4^ cells/well. We counted the cells 48 hours post-treatment using Trypan Blue dye to estimate both living and dead cells. To assess the effect of gadolinium (Gd^3+^) on ANE-induced cell growth, cells were pre-incubated with or without 1 µM Gd^3+^ 15 min prior to the addition of AN extracts and throughout the overnight incubation.

## Results

### Areca nut extracts, but not other Betel nut ingredients, mobilize calcium in immune cells

**Figure 1:**
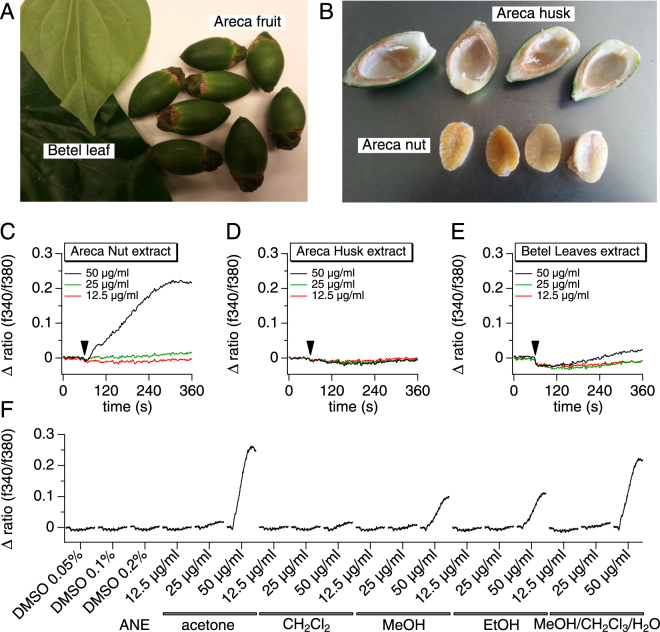
Extraction and identification of bio-active betel nut components. (**A**) Betel nut elements include the betel leaves and the actual Areca fruits (**A**) and the Areca fruit is composed of Areca nut kernel and the husk **(B)**. **(C–F)** RBL-2H3 cells were assayed using Fura-2-AM dye to measure a intracellular changes in calcium concentration following the application of different ingredients, extracted with a mixture of methanol, chloroform and water. Total Areca nut **(C)**, Areca husk **(D)** and betel leaves **(E)** extracts were applied at 12.5, 25 and 50 µg/ml to RBL-2H3 in standard 2 mM Ca^2+^ Ringer solution. The arrow indicates the time (60 s) of addition of the extracts. The calcium responses reflected by the change in fluorescence ratio (∆ ratio (340/380)) were recorded for an additional 300 seconds after application. **(F)** Areca Nuts were extracted using various solvents in order to compare the responses and determine the optimal conditions that allow a better yield of the active components. The extracts were then tested on RBL-2H3 using the same protocol as in (**C**–**E**). The traces were plotted in a serial and more condensed manner for comparison.

Areca nuts are consumed in various forms and come in preparations. They can be chewed alone (kernels with or without husks) or more often wrapped in betel leaves (*Piper betle L*.) in what is known as Betel Nut (BN) or by including other additives in the BN, such as slaked lime and tobacco or spices, also known as Betel quid^32^. To assess the role of BN in mobilizing cellular Ca^2+^, we exposed the rat mast cell line RBL-2H3 to extracts of various BN ingredients (Fig. 1A–B). Individual Areca nut components (kernels and husks) as well as betel leaves were incubated in a mixture of chloroform (CHCl_3_), methanol (MeOH) and water (12:5:3). The resulting extracts were filtered, dried, and dissolved in DMSO. The extract was diluted to final concentrations of 12.5, 25 and 50 µg/ml in standard Ringer’s solution containing 2 mM Ca^2+^. Figure 1 demonstrates that Areca Nut Extract (ANE) increased intracellular Ca^2+^ concentrations in RBL-2H3 cells at 50 µg/ml (Fig. 1C), while Areca husk and betel leaves extracts failed to induce any significant Ca^2+^ signals at the same concentration (Fig. 1D–E). This finding indicates that BN is indeed capable of mobilizing calcium signals in immune cells and that the Ca^2+^-activating components are present solely in the Areca nut kernels.

We then set out to screen multiple solvents in order to optimize the extraction protocol of Areca nuts. We tested acetone, dichloromethane (CHCl_2_), methanol (MeOH), ethanol (EtOH) and compared them to the signals obtained from the use of a mixture of chloroform, methanol and water. The resulting extracts were then tested for calcium mobilization activity and we found that both ethanol and methanol extracts resulted in significant Ca^2+^ signals. However, acetone (2.5-fold higher signal) and methanol/chloroform/water (2-fold higher signal) proved more potent, while dichloromethane extract was inactive (Fig. 1F). As a result of this screening, acetone was used as the standard solvent in the extraction protocol throughout the entire study.

### Areca nut extract induces both calcium release and calcium entry and the activity resides in the aqueous fraction

**Figure 2:**
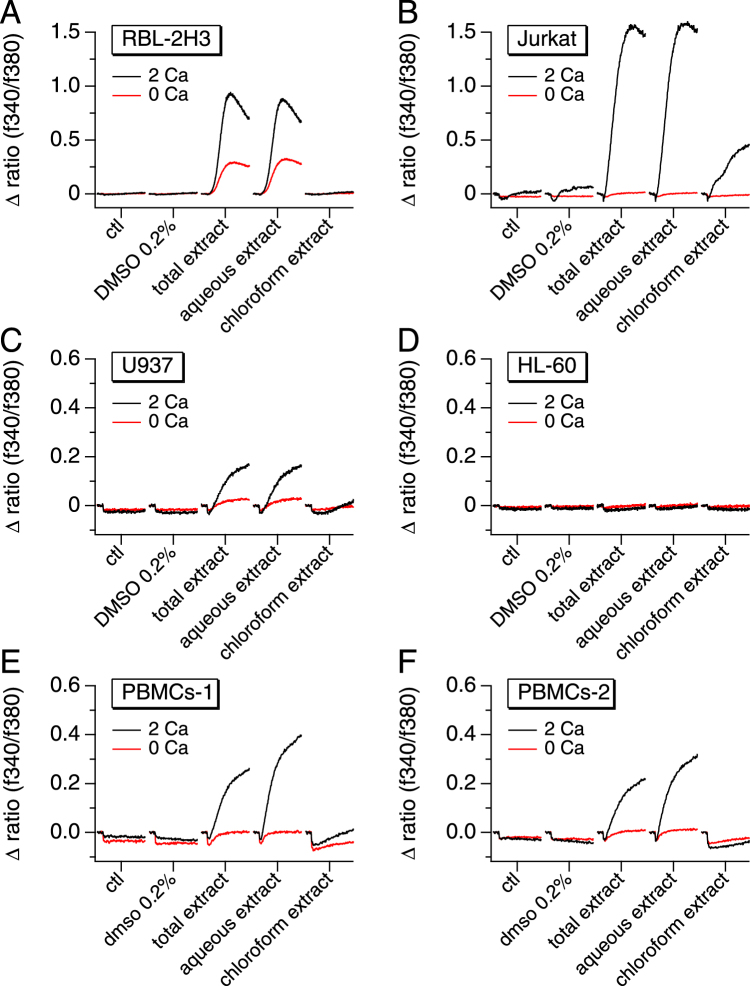
Areca nut-induced calcium responses are limited to the aqueous fraction and engender both calcium release and calcium entry. Average [Ca^2+^]_*i*_ responses before and following the application of 50 µg/ml Areca nut’s total, aqueous or organic (chloroform) extract in the presence (2 Ca) or absence (0 Ca) of external 2 mM CaCl_2_. The measurements were performed using RBL-2H3 rat mast cells (**A**), Jurkat human T cells (**B**), U937 human monocytes (**C**), HL-60 human neutrophils (**D**) and human primary Peripheral Blood Mononuclear Cells (PBMCs) from two different sources; PBMCs-1 from Lonza (**E**) and PBMCs-2 from Stemcell Technologies (**F**).

Given its stimulating effect on calcium signals, and in order to determine the nature of the active compounds, the whole Areca nut extract was further processed to separate the water-soluble components from the organic (chloroform) soluble ones. To assess their efficacy on mobilizing Ca^2+^, various immune cell lines were exposed to the whole ANE as well its aqueous and chloroform extracts. Figure 2 illustrates that ANE increased intracellular Ca^2+^ concentrations in the rat mast cell line RBL-2H3 (Fig. 2A), the human T lymphocyte line Jurkat (Fig. 2B), the human monocytic cell line U937 (Fig. 2C), but not in the human neutrophil-like cell line HL-60 (Fig. 2D). These data indicate that ANE components are capable of mobilizing Ca^2+^ in various important pro-inflammatory immune cell types. Surprisingly, HL-60 cells were unresponsive, suggesting that they may lack the signaling mechanism responsible for the Ca^2+^ mobilization observed in human primary neutrophils^30^. To verify that the observed response in these cell lines is representative of primary human cells, we performed similar Ca^2+^ measurements on human primary peripheral blood mononuclear cells (PBMCs). For a more precise measurement and reproducibility assessment, we obtained these primary cells from two different sources, as described in the materials and methods section. Interestingly, not only did both PBMC sets produce nearly identical calcium signals, but also induced a similar response profile as that observed in RBL-2H3, Jurkat and U937 (Fig. 2E–F). Our results also show that the Ca^2+^-mobilizing activity in ANE is induced by water-soluble compounds, as demonstrated by the generation of a comparable Ca^2+^ signal when the cells were perfused with the aqueous extract while the chloroform extract showed little to no effect.

In order to determine the source of calcium, the extracts were diluted to a final concentration of 50 µg/ml in standard Ringer’s solution containing 0 or 2 mM Ca^2+^ when applied to the cells. Figure 2 shows that both intracellular release and entry of extracellular Ca^2+^ contribute to the ANE-induced calcium signal. Moreover, the observed differences in the shapes and amplitudes of the Ca^2+^ signals may be indicative of the involvement of several mechanisms or simply to the calcium signature characterizing each cell type, as observed with the thapsigargin responses illustrated in supplementary Figure 1 (Sup. F. 1A–F).

### Ca^2+^ signals are not caused by various known Areca nut components

**Figure 3:**
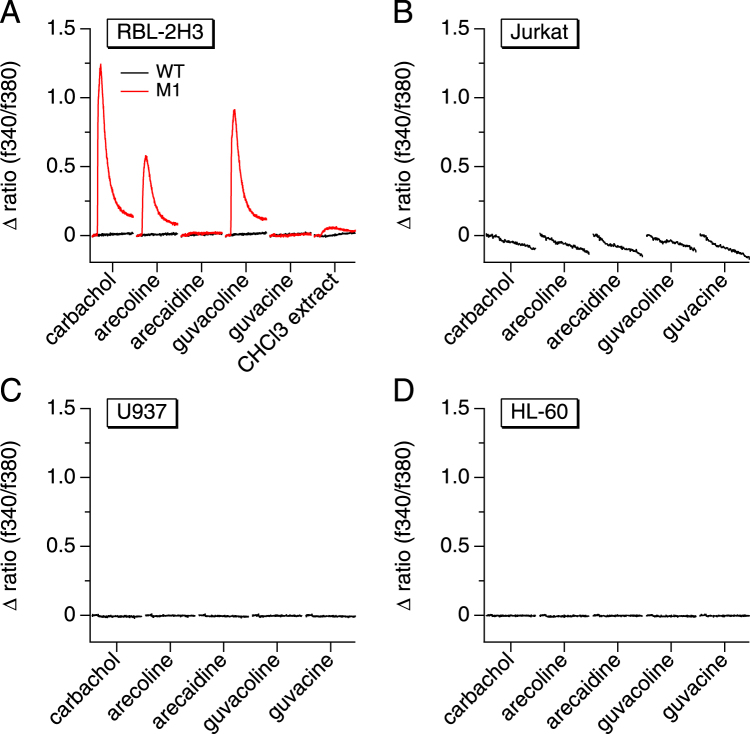
The effect of Areca nut alkaloids on calcium signals in immune cells. The graphs represent average [Ca^2+^]_*i*_ responses before and following the application of 100 µM arecoline, arecaidine, guvacoline and guvacine. The measurements were performed using the following immune cell lines: RBL-2H3 rat mast cells, Wild-Type (WT) and cells overexpressing the muscarinic acetylcholine receptor M1 (**A**), Jurkat human T cells (**B**), U937 human monocytes (**C**) and HL-60 human neutrophils (**D**). Carbachol was used at 100 µM as a positive control of the muscarinic response and the chloroform extract at 50 µg/ml as the main source of alkaloids.

While whole ANE can mobilize Ca^2+^, none of the major known Areca nut components have been shown to do so in immune cells. Among the best known chemical constituents of Areca nuts, four major alkaloids (arecaidine, arecoline, guvacine and guvacoline) have been identified^4,33^. The only known potential Ca^2+^-mobilizing components of Areca are arecoline and guvacoline, agonists of muscarinic receptors^34^, although their effects on Ca^2+^ signaling has not been reported and is unlikely to account for Ca^2+^ signals observed in immune cells as they do not express M1 or M3 receptors. Furthermore, the alkaloids are mostly partitioned in the organic extracts with little if any representation in the aqueous fraction. Nevertheless, we performed Ca^2+^-imaging experiments with these alkaloids to assess their ability to mobilize Ca^2+^ in RBL-2H3, Jurkat, U937, and HL-60 cells. As expected, none of the alkaloids, tested at 100 µM, caused any significant Ca^2+^ signals (Fig. 3A–D). This excludes these alkaloids from being responsible for the Ca^2+^ elevation induced by ANE. In control experiments, we tested RBL-2H3 overexpressing the muscarinic acetylcholine receptor M1 (RBL-M1) against the four alkaloids in comparison with the muscarinic cholinergic agonist carbachol. As expected, RBL-M1 were responsive to 100 µM carbachol but also to arecoline and guvacoline, which are both known to act as agonists of muscarinic receptors (Fig. 3A). As expected, RBL-M1 also showed a small Ca^2+^ response when perfused with the chloroform extract. The response observed in the organic fraction is likely caused by arecoline, the most abundant Areca nut alkaloid, typically constituting 0.3–0.63% of the dry weight of the nut^35^.

**Figure 4:**
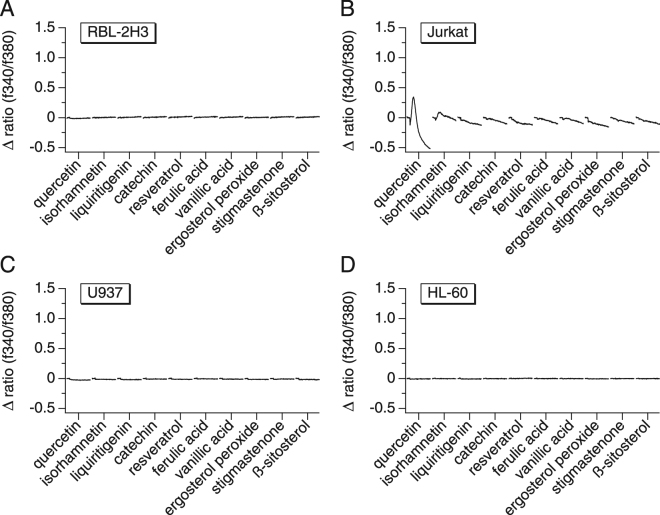
The effect of known Areca nut molecules on calcium signals in immune cells. The graphs represent average [Ca^2+^]_*i*_ responses before and following the application of the compounds at 10 µM. The measurements were performed using the following immune cell lines: RBL-2H3 rat mast cells (**A**), Jurkat human T cells (**B**), U937 human monocytes (**C**) and HL-60 human neutrophils (**D**).

After eliminating the alkaloids as the constituents responsible for inducing the observed Ca^2+^ signals, we considered and tested other chemical constituents that have been identified in Areca nuts. Yang *et al*. have described thirteen compounds, mainly flavonoids and sterols, that they have isolated from a 95% ethanol extract of the fruits of *Areca catechu*^36^. Their structures were identified as quercetin, isorhamnetin, liquiritigenin, (+)-catechin, resveratrol, ferulic acid, vanillic acid, 5,8-epidioxiergosta-6,22-dien-3beta-ol (ergosterol peroxide), stigmasta-4-en-3-one, ß-sitosterol, 5,7,4′-trihydroxy-3′,5′-dimethoxyflavanone, cycloartenol, and de-O-methyllasiodiplodin. We were able to test the commercially available compounds at a concentration of 10 µM and found that none of them were able to induce a calcium signal that is similar to one induced by ANE (Fig. 4A–D). Quercetin showed a small biphasic change in Ca^2+^ with a transient initial spike followed by a decrease. Quercetin has been shown to cause biphasic Ca^2+^ signals in Jurkat cells owing to its intracellular binding to proteins and its ability to release Ca^2+^ from intracellular stores.

Taken together, these results suggest that water-soluble components from the Areca nut may activate immune cells and be important players in the chronic inflammation and possibly carcinogenesis caused by betel nut chewing. In addition, the active constituents responsible might represent novel chemical structures as demonstrated by excluding Areca nut alkaloids and other known chemical components.

### The Ca^2+^-mobilizing AN extracts induce cytokine release and cell growth independently of SOCE

**Figure 5:**
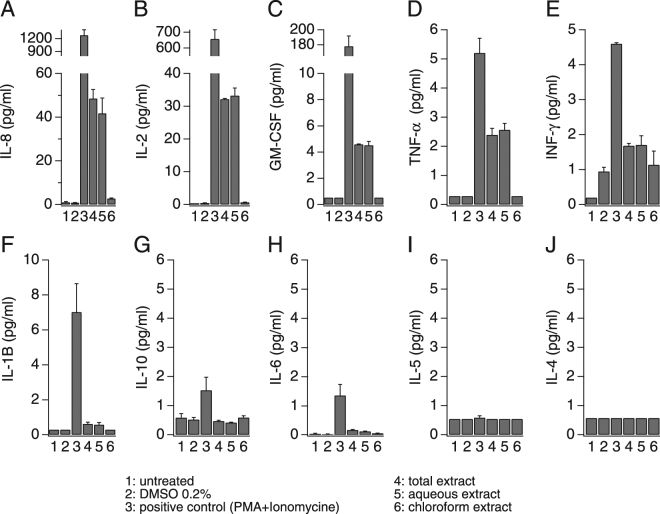
Areca nut extracts effect on cytokine release in Jurkat T cells. Cells were seeded at a density of 2 million cells/ml, stimulated for 24 h and then supernatants were analyzed for cytokines content. A combination of 50 ng/ml phorbol 12-myristate 13-acetate (PMA) and 1 µM ionomycin was used as positive control stimulus.

**Figure 6:**
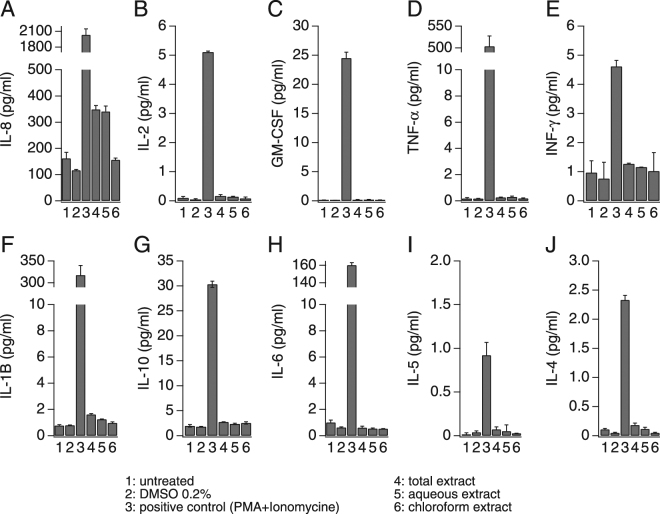
Areca nut extracts effect on cytokine release in U937 cells. Cells were seeded at the density of 2 million cells/ml, stimulated for 24 h and then supernatants were analyzed for cytokines content. A combination of 10 ng/ml phorbol 12-myristate 13-acetate (PMA) and 20 µg/ml lipopolysaccharides (LPS) was used as positive control stimulus.

**Figure 7:**
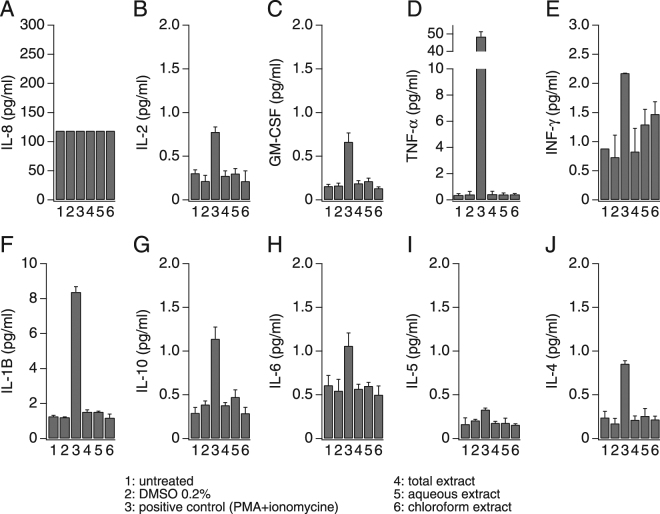
Areca nut extracts effect on cytokine release in HL-60 cells. Cells were seeded at the density of 2 million cells/ml, stimulated for 24 h and then supernatants were analyzed for cytokine content. 10 ng/ml phorbol 12-myristate 13-acetate (PMA) was used as a positive control stimulus.

The role of calcium as a second messenger in many cell types and pathways is well known and it is of particular importance in the maturation and activation of immune cells^37^. A chief mechanism of cytokine release in immune cells is through receptor-induced calcium influx. The resulting increase in intracellular Ca^2+^ concentration is both necessary and sufficient to elicit the release of certain pro-inflammatory mediators from neutrophils, monocytes, mast cells, and lymphocytes^38–40^. To determine whether AN extracts induce mediator release, we measured pro-inflammatory cytokines produced by the immune cell lines under investigation to assess the relative contribution and impact of the ANE-induced calcium signals on cytokine release. We used the high-throughput multi-analyte bead-based immunoassay (Luminex® technology) to quantify 10 different cytokines (GM-CSF, IFN-γ, IL-1β, IL-2, IL-4, IL-5, IL-6, IL-8, IL-10 and TNF-α). In analogy to the calcium assays, we tested the whole Areca nut extract as well as the aqueous and chloroform extracts and the resulting cytokine release under these conditions were compared to the results from untreated cells and cells treated with DMSO at a final concentration of 0.2% (vehicle). Three positive controls were used depending on the cell line: Jurkat cells were stimulated with combination of 50 ng/ml *phorbol* 12-myristate 13-acetate (PMA) and 1 µM ionomycin (Fig. 5), U937 were stimulated with 10 ng/ml PMA and 20 µg/ml Lipopolysaccharides (LPS) (Fig. 6) and finally HL-60 were stimulated with 10 ng/ml PMA (Fig. 7). As shown in Fig. 5, Jurkat cells release mainly IL-8, IL-2, GM-CSF and small amounts (4-5 pg/ml) of TNF-α, INF-γ and IL-1B. Interestingly, the total ANE was able to induce moderate amounts of IL-8 and IL-2 as well as small amounts of GM-CSF and TNF-α. The cytokine release results seem to correlate with the calcium activity, since only the total ANE and aqueous extract were able to increase the production of these cytokines, while the chloroform extract was inactive and showed similar basal activity as in the non stimulated cells (Fig. 5). Unlike Jurkat cells, only IL-8 release was induced by the AN extracts in U937, even though other cytokines were released by this cell line using PMA + LPS (Fig. 6). The correlation between calcium signaling and cytokine release was further confirmed by the absence of any effect of the chloroform extract on the IL-8 production in U937 (Fig. 6) as well as the absence of any cytokine release following AN extracts exposure in the non-responsive HL-60 (Fig. 7).

**Figure 8:**
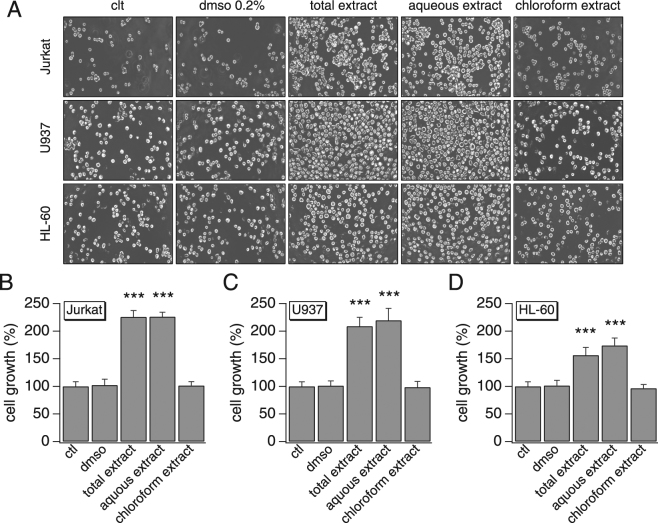
Effect of ANE-induced cytokine release on cell growth. Jurkat cells were treated with various AN extracts (50 µg/ml) and their conditioned medium was used to estimate the effect of the released amounts of cytokines on cell growth. (**A**) Cell growth images of different immune cell lines observed at 48 h post-treatment by an inverted microscope (total magnification, x100). (**B**) Average cell growth of Jurkat cells, normalized to the control. (**C**) Average cell growth of U937 cells, normalized to the control. (**D**) Average cell growth of HL-60 cells, normalized to the control. The values are mean ± SEM. The growth assay was repeated in 3 independent experiments where each condition was run in triplicate.

We then examined whether the secreted amounts of cytokines were sufficient to enhance cell growth. We pre-stimulated Jurkat cells with different AN extracts then used their conditioned medium to run a growth assay on all 3 immune cell lines (Jurkat, U937 and HL-60). Figure 8 shows that conditioned medium with total or aqueous AN extracts similarly increased cell proliferation of all 3 cells lines (1.6–2.3 fold, compared to the controls) while the chloroform extract condition remained comparable to the control conditions (untreated and DMSO-treated cells). No cell death was detected under any of the conditions. These results correlate with the effects we observed on cytokine release and represent a supporting evidence of AN role in the activation of immune cells and pro-inflammation.

**Figure 9:**
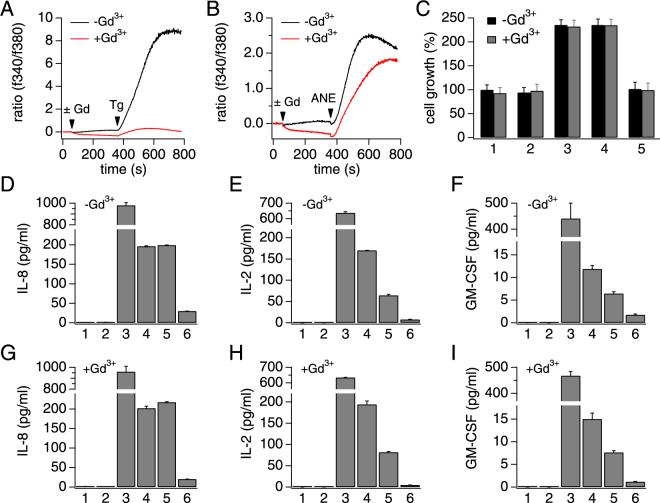
SOCE role in ANE-induced calcium signal and T cell activation. (**A**) A typical thapsigargin-induced SOCE signal in Jurkat cells and its full inhibition by 1 µM gadolinium (Gd^3+^). Thapsigargin was applied at 1 µM. (**B**) The effect of Gd^3+^ pre-incubation on the ANE-induced calcium response. AN total extract was used at 50 µg/ml. (**C**) Average cell growth of Jurkat cells, normalized to the control in the presence or absence of 1 µM Gd^3+^. Cell growth was assessed similarly to Fig. 8. 1: untreated, 2: DMSO 0.2%, 3: AN total extract, 4: AN aqueous extract and 5: AN chloroform extract. (**D–F**) Areca nut extracts effect on cytokine release in Jurkat T cells in the absence of Gd^3+^. (**E–I**) Areca nut extracts effect on cytokine release in Jurkat T cells in the presence of Gd^3+^. (**D–I**) 1: untreated, 2: DMSO 0.2%, 3: positive control (10 ng/ml PMA + 1 µM ionomycin), 4: AN total extract, 5: AN aqueous extract and 6: AN chloroform extract.

Store operated calcium entry (SOCE) is a well-characterized mechanism in many immune cells, particularly as an immediate downstream signaling component of T cell receptor (TCR) activation in lymphocytes^41–44^. We set out to determine whether or not ANE-induced calcium signaling and immune cells activation was mediated by SOCE. Since SOCE is inhibited by gadolinium (Gd^3+^) at low concentrations^45^, we used this pharmacological tool to assess the involvement of SOCE in ANE-induced calcium responses. We first confirmed that 1 µM Gd^3+^ completely inhibited thapsigargin-induced (1 µM) SOCE in Jurkat cells (Fig. 9A). However, the same concentration of Gd^3+^ only partially affected ANE-induced calcium response (Fig. 9B). We further assessed whether the partial calcium inhibition would translate in terms of T cell activation by testing Gd^3+^ on both cell growth and cytokine release. Our data show that pharmacological inhibition of SOCE did not prevent ANE from inducing cell growth or cytokine release (Fig. 9C–I). These findings indicate that the mechanisms responsible for the calcium signals and T cell activation in response to ANE exposure may be more complex than anticipated and possibly involve more than one target. Further purification of the active ingredients of ANE will help elucidate the signaling pathways involved.

## Discussion

The widespread habit of Areca chewing is associated with a high prevalence of oral carcinoma and numerous other diseases and represents an important global public health issue. Although several studies have analyzed the chemical composition of ANE and some alkaloids have been identified as candidates for the deleterious health effects on betel nut chewers, little is known about their effects on Ca^2+^ signaling and the early causative events of inflammation. It is also unknown what type of immune cells Areca nut chewing can affect. Our results demonstrate that ANE can induce calcium signals in at least 3 immune cell lines and human primary immune cells (PBMCs), inducing the production of pro-inflammatory cytokines. Further separation of the PBMCs into T cells, B cells and monocytes would potentially be of interest in elucidating specific responses to each cell subtype. We show that ANE effects engender calcium release from intracellular stores but mostly activates calcium influx. We further demonstrate that none of the established and well-known ANE compounds seem to be the Ca^2+^-mobilizing component, suggesting that the calcium-mobilizing pro-inflammatory molecules contained in Areca nut are novel and yet to be determined.

It is well known that Ca^2+^ signaling mechanisms are at the center of cellular responses in practically every immune cell type. Increasing or decreasing extracellular or cytosolic Ca^2+^ levels respectively enhance or prevents immune cell activation, cytokine production, and proliferation^38–41^. This highlights the immense importance of Ca^2+^ signaling in immune responses and hence every cell type has developed specific mechanisms to control Ca^2+^ release and Ca^2+^ entry. The most important and prevalent mechanism of Ca^2+^ mobilization in immune cells occurs through store-operated Ca^2+^ entry (SOCE), where highly Ca^2+^-selective channels known as CRAC (calcium release-activated calcium) channels can sustain long-lasting increases in intracellular Ca^2+^ ^46–50^. CRAC channels are store-operated channels that open when the endoplasmic reticulum (ER) Ca^2+^ store becomes depleted upon InsP_3_-induced Ca^2+^ release. All of the cell types investigated in the present study have been shown to utilize SOCE as a mechanism to mobilize calcium and support the production and release of cytokines. However, complete inhibition of SOCE by gadolinium only partially affected the ANE-induced calcium entry and T cell activation, suggesting that other calcium mobilization mechanisms might also be involved. While CRAC channels are the best characterized calcium channels and a critical calcium source in TCR signaling, other channels may contribute to calcium influx in T cells^42–44,51^. It is unlikely that the primary mechanism of initiating the calcium mobilization is caused by a thapsigargin-like inhibition of SERCA pumps, since Tg-induced activation of SOCE is observed in a large number of cell types, including cells in which ANE remains ineffective (e.g. HL-60 or HEK-293 cells). One possible mechanism might be the activation of yet to be identified surface receptors common to the responsive cell types.

Several Ca^2+^-permeable cation channels as well as Ca^2+^-impermeable Na^+^, K^+^, and Cl^−^ have been described in immune cells and may participate in shaping Ca^2+^ signals either through direct Ca^2+^ entry or by setting the driving force for Ca^2+^ entry through changes in membrane potential^48–50^. In addition to these channels, some immune cells (e.g., neutrophils, monocytes, and macrophages) also utilize TRPM2, a member of the melastatin-related transient receptor potential (TRPM) family of cation channels, for Ca^2+^ signaling^51–55^. TRPM2 has gained importance in inflammation, as it can be activated by reactive oxygen species and serve both as plasma-membrane Ca^2+^ entry pathway as well as a lysosomal Ca^2+^ release channel^56–59^. In the context of the cells investigated here, TRPM2 is found to be expressed in U937^56,60^, HL-60^61,62^, and Jurkat cells^63,64^ but not in RBL-2H3 (unpublished observations). Given the use of crude extracts, which are composed of a mixture of molecules, it is also possible that we are activating multiple synergistic or compensatory calcium mechanisms and inhibiting a single source of calcium would not be the proper way to unveil the full signaling pathway. Unless we successfully purify the active ingredients, it would be challenging to accurately characterize the exact pro-inflammatory mechanisms of Areca nut.

Our results have also highlighted the role of Areca nut in the production of some inflammatory cytokines that lead to increased cell proliferation, particularly IL-8 that was abundantly released in both T cells and monocytes. Unlike many other cytokines, IL-8 has a very distinct target specificity for neutrophils, suggesting that these cells might still have a crucial role in the inflammatory diseases induced by Areca nut as indicated by our growth assay results in Fig. 8D, even though they were non-responsive to direct exposure to AN extracts^65,66^. IL-8 is a unique cytokine that also plays an important role in cancer growth and is secreted by oral squamous cancer cells^67^. Additionally, studies that investigated saliva as a diagnostic biofluid, suggested IL-8 as a biomarker for oral squamous cell carcinoma and chronic oral inflammatory diseases^68,69^. Although we only tested immune cell lines in this study, it is possible that ANE may also induce IL-8 secretion in oral epithelial cells, amplifying the inflammatory effects of AN chewing and contributing to the initiation and/or progression of oral cancer.

## Electronic supplementary material

Supplementary Figure 1
