# Supplementary material for: Areca nut extracts mobilize calcium and release pro-inflammatory cytokines from various immune cells
Source: Sci Rep. 2018 Jan 18;8:1075. doi: 10.1038/s41598-017-18996-2 (PMC5773534; doi:10.1038/s41598-017-18996-2)
Supplement: Supplementary file 1 — Supplementary Figure 1 [file 41598_2017_18996_MOESM1_ESM.pdf]

Areca nut extracts mobilize calcium and release pro-inflammatory cytokines from various immune cells

Malika Faouzi[1*], Ram P. Neupane[2], Jian Yang[3], Philip Williams[2], Reinhold Penner[1,4*]

[1] Center for Biomedical Research, The Queen's Medical Center, Honolulu, HI 96813

[2] Dept. of Chemistry, University of Hawaii, Honolulu, HI 96822

[3] College of Natural and Applied Sciences, University of Guam, Mangilao, Guam 96923

[4] Cancer Biology Program, University of Hawaii Cancer Center, Honolulu, HI 96813

* Correspondence should be addressed to: Reinhold Penner or Malika Faouzi, The Queen's Medical Center, Center for Biomedical Research, 1301 Punchbowl St., Honolulu, HI 96813, U.S.A. e-mails: rpenner@hawaii.edu, mfaouzi@hawaii.edu

Supplementary Figure 1

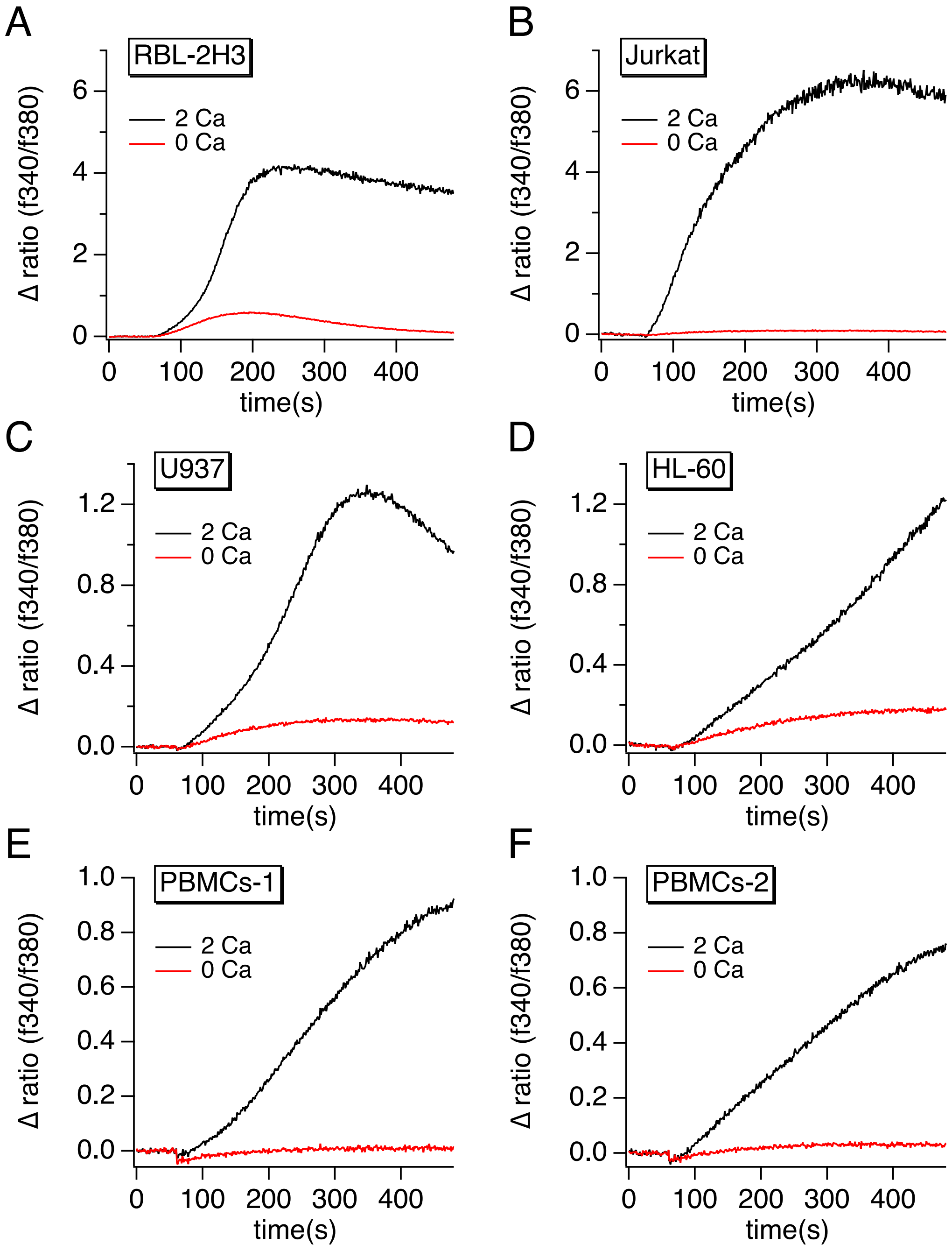

Supplementary Figure 1: SOCE signature in various immune cells. Thapsigargin-induced SOCE signals in RBL-2H3 rat mast cells **(A)**, Jurkat human T cells **(B)**, U937 human monocytes **(C),** HL-60 human neutrophils **(D)** and human primary Peripheral Blood Mononuclear Cells (PBMCs) from two different sources; PBMCs-1 from Lonza **(E)** and PBMCs-2 from Stemcell Technologies **(F)**. The signals were recorded in the

presence (2 Ca) or absence (0 Ca) of 2 mM external calcium, representing SOCE and calcium release signals, respectively.
